# Post–Critical Illness Dysphagia in the Intensive Care Unit (the Dysphagia-ICU Study): Protocol for a Prospective Cohort Study

**DOI:** 10.2196/68053

**Published:** 2025-08-14

**Authors:** Waleed Alhazzani, Kimberley Lewis, Jamala Selan, Haifa F Alotaibi, Sara Alnasser, Afaf Alrwais, Atheer Alhomoud, Rawabi M Alsayer, Hani Tamim

**Affiliations:** 1 Health Research Center Ministry of Defense Health Services Riyadh Saudi Arabia; 2 Critical Care Department College of Medicine King Saud University Riyadh Saudi Arabia; 3 Department of Medicine McMaster University Hamilton, ON Canada; 4 Communication and Swallowing Disorders Division ORL Head and Neck Surgery Prince Sultan Military Medical City Riyadh Saudi Arabia; 5 College of Medicine Alfaisal University Riyadh Saudi Arabia; 6 Clinical Research Institute Department of Medicine American University of Beirut Medical Center Beirut Lebanon

**Keywords:** Dysphagia, intensive care, post–critical illness dysphagia, protocol, cohort study

## Abstract

**Background:**

Post–critical illness dysphagia occurs in about 10% to 62% of patients in the intensive care unit (ICU). Studies focusing on risk factors for dysphagia following endotracheal intubation are scarce and provide conflicting results. More research is required to determine the true prevalence of dysphagia, possible mechanisms, and risk factors.

**Objective:**

The aims of the study are to determine (1) risk factors associated with post–critical illness dysphagia, (2) the prevalence of post–critical illness dysphagia, (3) outcomes of patients with post–critical illness dysphagia, and (4) the diagnostic accuracy of the water sip test compared to fiber-optic endoscopic evaluation of swallowing (FEES).

**Methods:**

We plan to undertake a single-center prospective cohort study of patients with post–critical illness dysphagia admitted to the ICU at Prince Sultan Military Medical City, Saudi Arabia. Our inclusion criteria are as follows: (1) being an adult (age 18 years and older), (2) having undergone invasive mechanical ventilation for >24 hours, (3) having been extubated for >24 hours, (4) being able to participate in a FEES, and (5) being hemodynamically stable. Each day, the research coordinator will screen all patients in the ICU for enrollment in the study. The research coordinator will collect daily data on the use of advanced life support, the need for mechanical ventilation, and outcomes (mortality, duration of mechanical ventilation, ICU length of stay, and hospital length of stay). Enrolled patients will undergo a water sip test in the ICU and FEES performed by a certified speech-language pathologist. Patients will then be followed during a hospital stay truncated for analysis at 30 days as two cohorts: (1) patients that have dysphagia, that is, abnormal FEES results; and (2) patients who have normal FEES results. Logistic regression will identify dysphagia risk factors (eg, age, Acute Physiology and Chronic Health Evaluation II score, and ventilation duration), while sensitivity and specificity tests will be used to compare the results of the water sip test to the FEES. Survival analysis will be used to evaluate 30-day outcomes.

**Results:**

The study anticipates enrolling 200 patients over 18 months. Key outcomes include dysphagia prevalence and risk factors. Final results will be analyzed and reported upon completion of the 30-day follow-up for all participants. As of July 13, 2025, a total of 45 patients had been enrolled, with an average recruitment rate of 7.5 patients per month.

**Conclusions:**

The study findings will promote early intervention for patients with post–critical illness dysphagia, improve multidisciplinary care, and inform policymakers for better resource allocation. This study lays the groundwork for future research and clinical guidelines tailored to Saudi Arabia’s health care context.

**International Registered Report Identifier (IRRID):**

PRR1-10.2196/68053

## Introduction

About 20 million patients are admitted to intensive care units (ICUs) globally each year. Those that survive frequently have post–critical illness complications that impair their quality of life [1]. Post–critical illness dysphagia is a well-recognized complication in the ICU [2]; however, it is an area that is under-studied [3]. Dysphagia refers to an impairment of the process of deglutition affecting the oral, pharyngeal, and/or esophageal phases of swallowing; it has been reported to occur in 10% to 62% of patients who undergo invasive mechanical ventilation in the ICU [2,4]. Dysphagia can persist for more than 6 months after critical illness [5].

The cause of post–critical illness dysphagia is unclear, but there are several hypotheses, such as motor and sensory impairment of the tongue [6]; laryngeal injury secondary to endotracheal intubation [7,8]; neuromuscular impairment associated with underlying comorbidities, neuromuscular blockade use, or critical illness neuropathy [5]; and cognitive impairment secondary to critical illness [9]. Post–critical illness dysphagia is associated with delayed oral intake, malnutrition, aspiration, prolonged hospitalization, and death [10,11].

However, several studies have reported that screening occurs in <50% of patients [12,13]. In clinical practice, the detection and management of post–critical illness dysphagia requires a multidisciplinary collaboration between physicians, nurses, speech language pathologists (SLPs), and physiotherapists. Unfortunately, there are no published high-quality studies to describe post–critical illness dysphagia in Saudi Arabia. Donohue et al [14] conducted a systematic review of 51 studies on oropharyngeal dysphagia in critically ill adults during acute and postacute recovery. They found a wide prevalence range—from 15% to 100%. Persistent dysphagia affected up to 74% at discharge and up to 22% at 10 to 17 months. Most studies had serious limitations: high risk of bias, inconsistent assessment tools, and varied outcomes. These flaws weakened the study’s conclusions. The authors called for prospective, longitudinal studies using standardized assessments to better define prevalence and long-term impact. Herein, we present a multidisciplinary research project to determine the prevalence and consequences of post–critical illness dysphagia in patients in Saudi Arabia.

The primary objective of this study is to determine the predictors associated with patient-important outcomes. The secondary objective is to determine the prevalence of post–critical illness dysphagia in the ICU of Prince Sultan Military Medical City, a tertiary care center in Saudi Arabia.

## Methods

### Study Design

**Figure 1:**
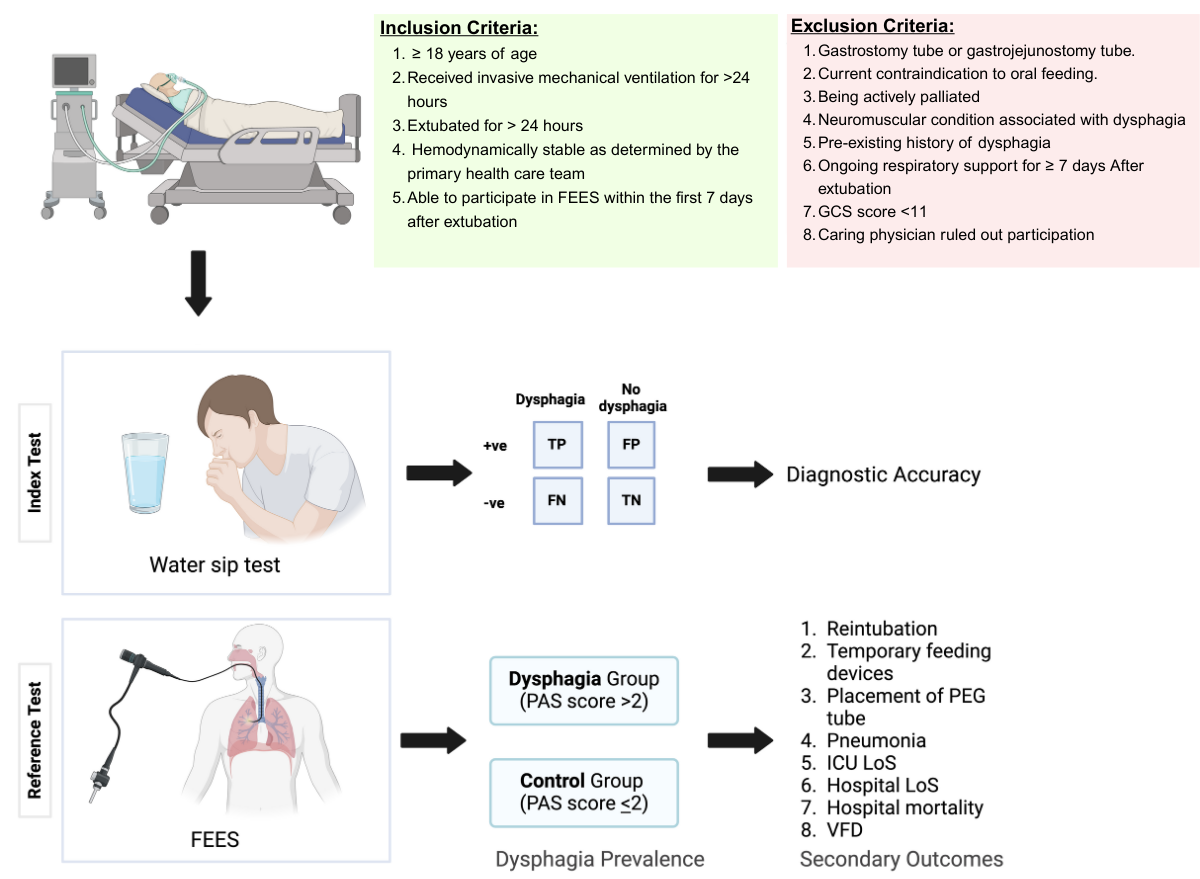
Summary of the Dysphagia-ICU Study.

This study will be a single-center prospective cohort study carried out in a tertiary care ICU (Prince Sultan Military Medical City) in Saudi Arabia. Recruitment will occur over 18 months, with follow-up truncated for analysis at 30 days after enrollment. Figure 1 illustrates the design of the study.

### Population

Eligible patients must meet the criteria shown in Textbox 1.

**Inclusion and exclusion criteria.**

**Inclusion criteria**

- Aged ≥18 years
- Received invasive mechanically ventilated for >24 hours
- Have been extubated for >24 hours
- Hemodynamically stable as determined by the primary health care team (ie, not requiring active resuscitation or vasoactive agents)
- Able to participate in fiber-optic endoscopic evaluation of swallowing within the first 7 days after extubation.

**Exclusion criteria**

- Gastrostomy tube or a gastrojejunostomy tube
- Current contraindication to oral feeding (eg, recent gastrointestinal surgeries as indicated by the surgeon)
- Being actively palliated
- Neuromuscular condition associated with dysphagia (eg, amyotrophic lateral sclerosis, myasthenia gravis)
- Preexisting history of dysphagia
- Ongoing respiratory support (defined as requiring ≥50% oxygen, bilevel positive airway pressure, or high-flow nasal cannula) for 7 days or more after extubation
- Glasgow Coma Scale score <11
- Caring physician ruled out participation

### Feasibility Assessment

We will assess feasibility outcomes after the first 50 patients are enrolled in the study. A successful consent rate will be defined as 70% of substitute decision makers (SDMs) or patients approached to consent choosing to participate in this study. In addition, a successful recruitment rate will be defined as achieving enrollment of 50 patients at a recruitment rate of 2 patients per screening week. Recruitment and consent rate will be reviewed weekly, and the screening records will be reviewed monthly with cases of missed eligible patients reviewed. If applicable, barriers to enrollment will be addressed to maximize recruitment. The recruitment metric will be measured and interpreted once 50 patients have been enrolled.

### Dysphagia Assessment

Enrolled patients will undergo a water sip test in the ICU, performed by the study SLP or the bedside nurse. First, the study SLP or bedside nurse will ask the patient to drink 5 ml of water (approximately 1 teaspoon) while sitting upright or at >30°. If the patient tolerates this small volume, the patient will then be asked to drink 90 ml of water from a cup, straw, or syringe. The study SLP or bedside nurse will observe the patient for 1 minute after drinking the water, looking for indirect signs of aspiration, such as coughing, grunting, or change in voice quality. The test is positive when these signs appear or when the patient cannot drink the full volume of water. Other volumes of water can also be used (2 × 5 ml, 2 × 10 ml, 2 × 20 ml, or 90 ml). The patient will be asked to produce the sustained vowel sound /a/ before and after swallowing the water to observe any voice change after swallowing. In addition, a certified study SLP will perform a FEES to assess swallowing. Although video fluoroscopy swallowing studies are another valid diagnostic tool [15,16], we elected to perform a FEES to minimize the risks associated with patient transfer. Previous studies have identified silent aspiration rates ranging upward from 25%, therefore, direct visualization using FEES is important. While the patient is in a sitting or upright position, the study SLP will use a disposable flexible fiber-optic rhinoscope directly passed through the nasal cavity using lubricated nonanesthetic gel down to the level of the soft palate or below. During FEES, the patient will be asked to swallow substances with a variety of consistencies (eg, thin fluids, thick fluids, and purees) colored with a food dye (blue or green) to help in visualization of aspiration and penetration in varying volumes (3 ml, 5 ml, and up to 10 ml). Although FEES is considered a safe procedure, study participants and/or their SDMs will be informed about potential complications during the consent process, which include discomfort, epistaxis (0.3% to 1.1%), swallowing inhalation, vasovagal episodes (6 in 1000), laryngospasm (3 in 1000) and mucosal laceration (a very rare complication). In this study, FEES serves as the reference standard for diagnosing dysphagia.

### Interpretation of the FEES

The study SLP will complete the Penetration Aspiration Scale (PAS) after completing the FEES for every patient and classify safety status for swallowing as safe (scores 1 or 2) or unsafe (scores above 2; Table 1) [17] The results will be collectively reported as positive (oropharyngeal dysphagia present, ie, an abnormal result) or negative (ie, a normal result). A PAS score >2 will be considered abnormal and indicative of oropharyngeal dysphagia. This cutoff is based on previously validated thresholds for aspiration risk.

**Table 1:** Penetration Aspiration Scale (PAS) score interpretation.

PAS score	Description	Safety status
1	Material does not enter the airway	Safe
2	Material enters the airway, remains above the VCs^a^, ejected from the airway	Safe
3	Material enters the airway, remains above the VCs, not ejected from the airway	Unsafe
4	Material enters the airway, contacts the VCs, ejected from the airway	Unsafe
5	Material enters the airway, contacts the VCs, not ejected from the airway	Unsafe
6	Material enters the airway, passes below the VCs, ejected from the trachea	Unsafe
7	Material enters the airway, passes below the VCs, not ejected despite effort	Unsafe
8	Material enters the airway, passes below the VCs, no effort made to eject	Unsafe

^a^VC: vocal cord.

### Ethical Considerations

This study has been reviewed and approved by the Central Research Ethics Committee of the Ministry of Defense Health Services, Riyadh, Saudi Arabia (H-01-R-096). The study protocol adheres to the ethical principles outlined in the Declaration of Helsinki and complies with local regulations governing research involving human participants.

Informed consent will be obtained from all participants or their legally authorized SDMs prior to enrollment. All collected data will be anonymized and stored securely in accordance with institutional policies to protect participant confidentiality.

The study research coordinator (RC) will screen all patients in the ICU during weekdays to avoid incurring additional weekend on-call costs. Due to the nature of their illnesses, associated life-sustaining treatments, use of sedatives and analgesic medications, and frequency of delirium, eligible patients may not be capable of participating directly in the informed consent process [18-20]. The study RC will approach patients who meet the eligibility criteria and can provide consent for written consent after extubation. However, as most patients will be incapable of consenting at the time of enrollment, the RC will approach the SDM to provide a priori consent. If the SDM is not available in person, the RC will use a telephone script to complete the consent procedure.

### Data Collection

The study RC will collect patient baseline data, including demographics, illness severity, need for advanced life support, and comorbidities. In addition, they will collect the results of the water sip test and FEES, outcomes (ie, dysphagia, reintubation events, pneumonia, mortality, percutaneous endoscopic gastrostomy tube insertion, and hospital and ICU length of stay), and source documentation that will help with the adjudication of outcomes. A patient’s vital status will be recorded at 30 days if the patient was not discharged from the hospital. Mortality, duration of ventilation, and ICU stay outcomes will be truncated for analysis at 30 days.

Data will be collected using electronic case report forms via the REDCap data capture system [21] All personal information, including informed consent forms and subject code lists, will be stored electronically on a secured network drive dedicated to the study that is located in a locked research room and accessible only by designated study staff.

### Outcomes

The clinical outcomes are the following (all truncated at 30 days after the FEES assessment):

- Dysphagia, defined as abnormal FEES scores resulting in the need for modification of oral diet texture or non-oral means of nutrition and hydration
- Dysphagia severity, ranging from severe (defined as an abnormal FEES requiring nothing per oral recommendation) to mild or moderate (defined as an abnormal FEES that requires any adjustment to diet texture)
- Reintubation, defined as reintubation within 30 days of the original extubation during initial hospital admission (reasons for reintubation will be recorded)
- Use of temporary feeding devices such as nasogastric or nasoduodenal feeding tubes
- Use of total parenteral nutrition
- Placement of a percutaneous gastrostomy feeding tube
- Pneumonia, defined as new pneumonia after extubation as determined by the treating health care team
- ICU length of stay, recorded as days from ICU admission to discharge from the ICU
- Hospital length of stay, recorded as days from ICU admission to hospital discharge
- Mortality during hospital stay, truncated for analysis at 30 days
- Ventilator-free days, defined as days alive and not receiving invasive mechanical ventilation at 30 days

### Statistical Analysis

#### Sample Size

To estimate the required sample size, we will assume a conservative prevalence of dysphagia of 35%; if we enroll 200 patients (anticipating 70 dysphagia events), this will allow for 6 covariates in a logistic regression model, including age, length of mechanical ventilation, Acute Physiology and Chronic Health Evaluation (APACHE) II score on admission, functional status defined by the Functional Status Score for the ICU (FSS-ICU), use of neuromuscular blocking agents, and use of systematic glucocorticoids.

#### Feasibility Assessment

After enrollment of 50 patients, we will assess feasibility outcomes. The consent rate will be calculated as the overall proportion of SDMs or patients consenting among those approached (with 95% CI), and the recruitment rate will be calculated as the mean number of recruited patients per active screening month. The target consent and recruitment rates are 70% and 8 patients per month, respectively.

#### Descriptive Statistics

Descriptive statistics will be used to analyze the baseline characteristics and will be reported as a count (percentage) for categorical variables and mean (SD) or median (IQR) for continuous variables, depending on the distribution. The prevalence of dysphagia will be computed as the number of patients with dysphagia divided by the number enrolled and reported as a percentage with 95% CIs.

#### Inferential Statistics

A bivariate analysis, such as the *χ*^2^ test or *t* test, will be applied to assess the association between each risk factor and the occurrence of dysphagia. Then, logistic regression will be used to determine the variables associated with post–critical illness dysphagia (defined a priori as length of mechanical ventilation, age, APACHE II score on admission, functional status defined by the FSS-ICU, use of neuromuscular blocking agents, and use of systematic glucocorticoids). Covariates will be entered into the model as a block. Model fit will be assessed using the Hosmer and Lemeshow goodness of fit statistic, with odds ratios and 95% CIs reported. A *P* value <.05 will be considered significant.

#### Diagnostic Accuracy

Sensitivity and specificity will be used to check dysphagia screening using a FEES, with the cutoff point for the dysphagia group being a PAS score >2. The sensitivity will present the proportion of true positive results (ie, correctly identified cases) among all patients who have dysphagia. In addition, the specificity will present the proportion of true negative results (ie, correctly identified noncases) among all the patients who do not have dysphagia. In this study, we will focus on sensitivity to avoid missing any positive cases and to determine how well the water sip test can correctly identify dysphagia. Moreover, a receiver operating characteristic curve analysis will be used, and the area under the curve will be calculated, to assess the overall performance of the water sip test.

#### Survival Analysis

Kaplan-Meier curves will be used to compare time-to-event outcomes (eg, dysphagia resolution, pneumonia, reintubation, or mortality) between patients with abnormal versus normal FEES results. Log-rank tests will assess differences in survival probabilities.

### Timeline

At a predicted recruitment rate of 2 patients per week, we anticipate that 18 months will be required to recruit 200 patients (Table 2).

**Table 2:** Study timeline and procedures.

Study procedures	Visit 1 (day 0): eligibility/enrollment (eCRF^a^); demographics (eCRF)	Visit 2 (days 1-7): FEES^b^ assessment; FEES results (eCRF); daily data collection	Visit 3 (days 2-29): daily data collection	Visit 4 (day 30; final study visit): ICU^c^/hospital discharge (eCRF)
Informed consent	✓			
Eligibility assessment	✓			
Demographics	✓			
Water sip test		✓		
FEES assessment		✓		
Outcome data collection		✓	✓	✓
Dysphagia		✓		
Aspiration		✓	✓	✓
Reintubation			✓	✓
PEG^d^ tube insertion			✓	✓
Pneumonia			✓	✓
ICU LOS^e^				✓
Hospital LOS				✓
Mortality (in ICU or in hospital)				✓

^a^eCRF: electronic case report form.

^b^FEES: fiber-optic endoscopic evaluation of swallowing.

^c^ICU: intensive care unit.

^d^PEG: percutaneous endoscopic gastrostomy.

^e^LOS: length of stay.

## Results

The Dysphagia-ICU study is currently in the active recruitment phase at Prince Sultan Military Medical City, Saudi Arabia. The study received institutional review board approval on September 25, 2024 (18-2024-04-08-93). As of July 13, 2025, a total of 45 patients had been enrolled. The current recruitment rate averages 7.5 per month, aligning with the projected pace to complete enrollment within the planned 18-month period. All participants to date have undergone the water sip test and FEES within 7 days of extubation, and no adverse events related to the FEES procedure have been reported.

## Discussion

### Principal Findings

Through implementation of the Dysphagia-ICU study, we aim to generate data on the prevalence of, risk factors for, and outcomes of post–critical illness dysphagia among critically ill patients in Saudi Arabia. Our anticipated main findings include identifying key predictors of post–critical illness dysphagia, determining the prevalence of dysphagia, evaluating the accuracy of bedside screening tools, and assessing the impact of dysphagia on patient outcomes such as ICU length of stay, pneumonia, reintubation, and mortality.

This study aims to establish the prevalence of post–critical illness dysphagia using FEES as the reference standard. We anticipate that post–critical illness dysphagia will be a significant yet underdiagnosed complication among ICU patients, as previous studies have relied on less sensitive bedside screening methods [2,3]. By identifying modifiable and nonmodifiable risk factors, this study will help guide early detection and intervention strategies [4,5].

Post–critical illness dysphagia remains underdiagnosed in ICUs, with its true prevalence often underestimated due to unreliable assessments [2,10]. While international data exist, studies in Saudi Arabia are limited. Dysphagia affects up to 62% of mechanically ventilated patients [4], but inconsistent evaluation methods impact prevalence estimates. Our Dysphagia-ICU study will use FEES for precise detection, bridging this gap by identifying prevalence, risk factors, and outcomes to inform clinical and policy decisions [11].

A key strength of this study is its comprehensive design, incorporating both clinical assessments and instrumental evaluations to accurately diagnose dysphagia. Additionally, the prospective cohort design will enable tracking of patient outcomes over 30 days, providing valuable insights into the short-term impact of post–critical illness dysphagia. However, as a single-center study, the generalizability of the findings may be limited. Future multicenter studies are needed to confirm these findings in a broader population.

Despite its limitations, this study promises to establish the foundation for regional guidelines. For patients, identifying dysphagia early can lead to prompt intervention, potentially reducing risks such as respiratory complications and malnutrition. For health care professionals, the study outcomes will strengthen multidisciplinary collaboration, enhancing the recognition and management of dysphagia. For policymakers, a clearer understanding of the burden posed by this condition will facilitate more effective resource allocation and health care planning in Saudi hospitals and ICUs, ultimately improving patient outcomes.

The results of the Dysphagia-ICU study will also lay the foundation for future research and the development of clinical practice guidelines tailored to the needs of the Saudi health care system.

### Conclusions

The study's findings will enhance early detection of dysphagia, thereby reducing complications, and provide insights for health care providers and policymakers to develop new guidelines for improving ICU care for critically ill patients.

## Abbreviations

APACHE: Acute Physiology And Chronic Health Evaluation
FEES: fiber-optic endoscopic evaluation of swallowing
FSS-ICU: Functional Status Score for the Intensive Care Unit
ICU: intensive care unit
PAS: Penetration Aspiration Scale
RC: research coordinator
SDM: substitute decision maker
SLP: speech-language pathologist

## Data Availability

The datasets generated or analyzed during this study are available from the corresponding author on reasonable request.
